# Supplementary material for: Auricular malformations are driven by copy number variations in a hierarchical enhancer cluster and a dominant enhancer recapitulates human pathogenesis
Source: Nat Commun. 2025 May 17;16:4598. doi: 10.1038/s41467-025-59735-w (PMC12085581; doi:10.1038/s41467-025-59735-w)
Supplement: Supplementary file 1 — Supplementary information [file 41467_2025_59735_MOESM1_ESM.pdf]

Supplementary Information

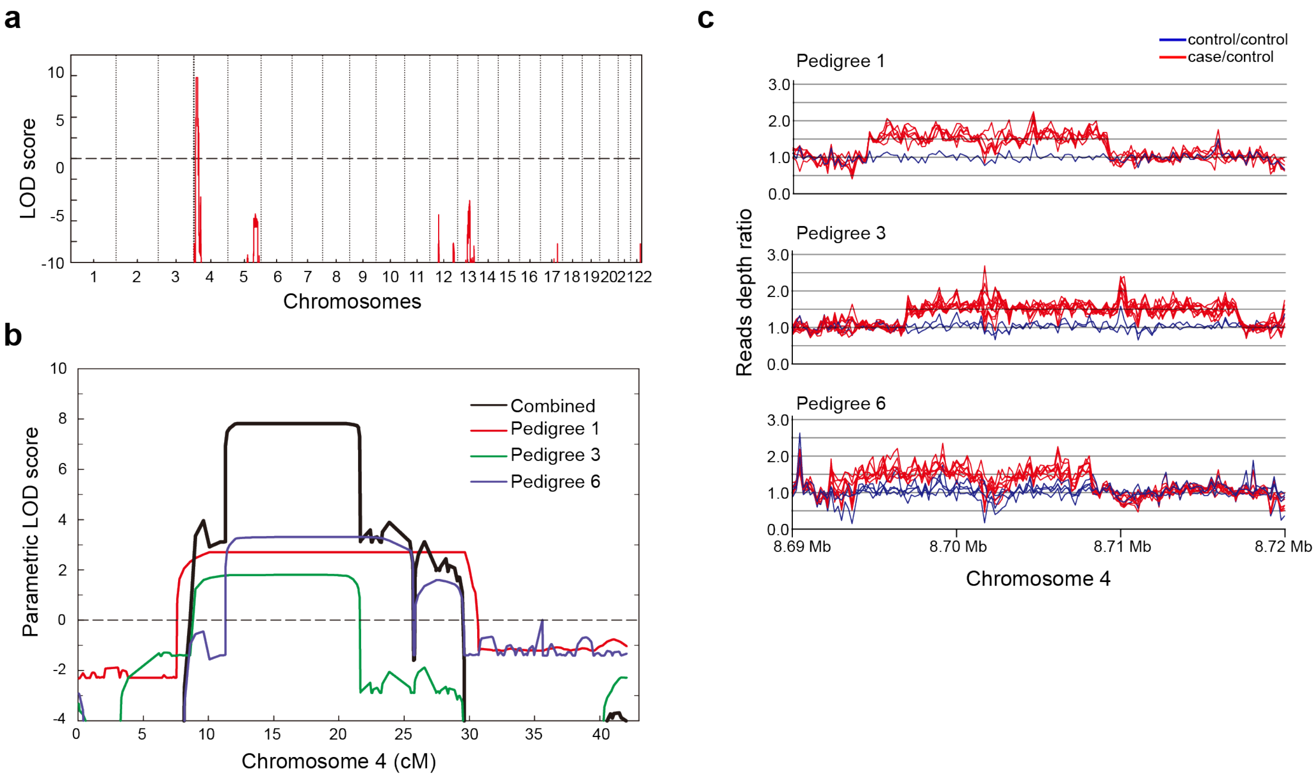

Supplementary Figure 1 | Identification and fine mapping of the BCE core locus in three BCE pedigrees.

a, Linkage analysis using whole-genome chip-based data in three BCE families revealed a pronounced signal on chromosome 4, marked by a LOD score of 7.8. This highlights a shared chromosomal region associated with the BCE phenotype. **b**, A detailed representation of the chromosome 4 linkage signal, including individual LOD scores for each family. This demonstrates the distinct yet overlapping linkage signals among these families. **c**, Fine mapping of the linkage signals identified copy number duplications through target capture sequencing. For each family, one healthy individual was randomly selected as a control. This panel compares the sequencing coverage depth ratio between the remaining family members and this healthy control. Red lines illustrate the read depth ratio in the linked chromosomal region for patients compared to the control, whereas blue lines depict the ratio between healthy family members and the control.

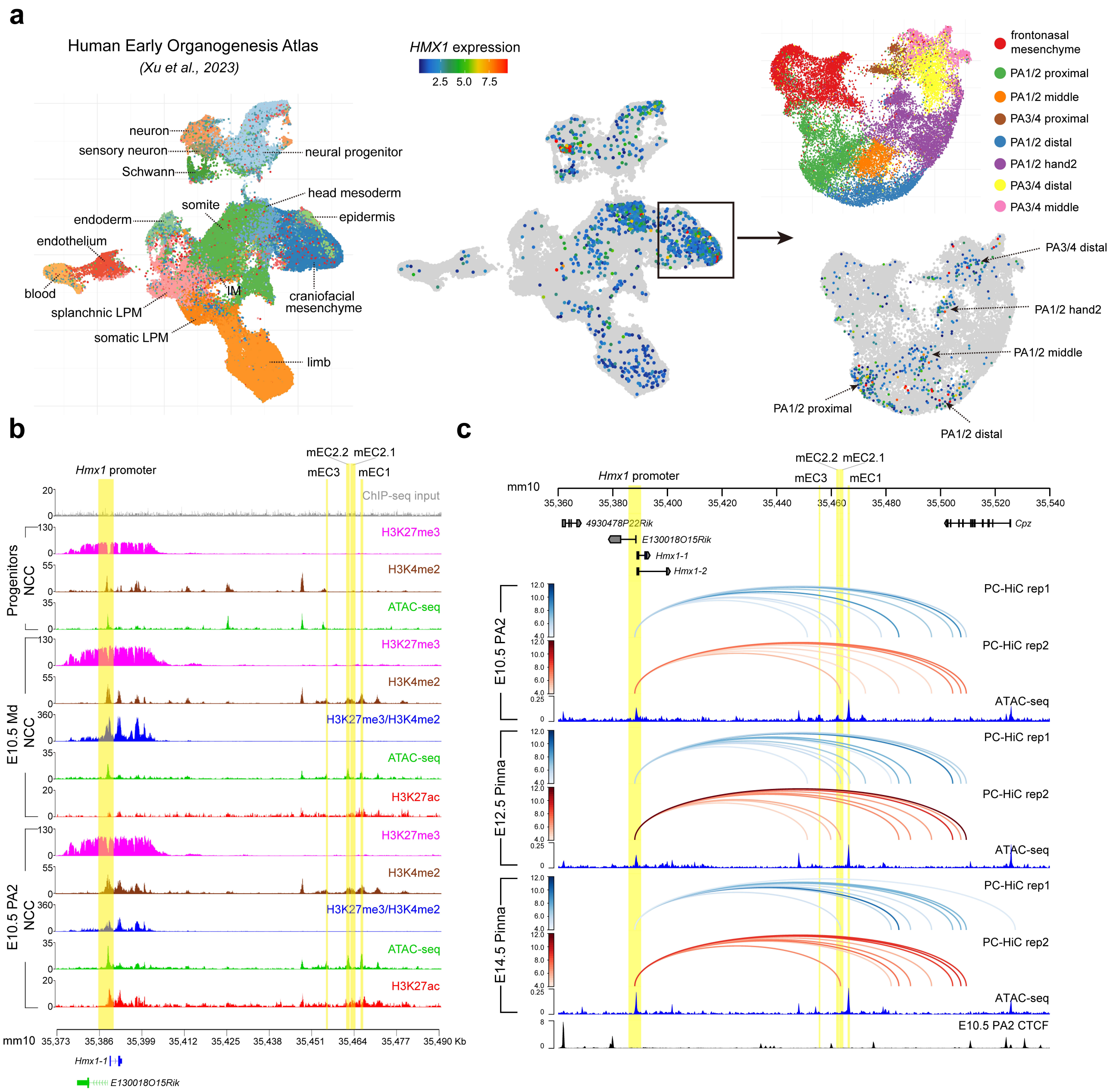

Supplementary Figure 2 | Single-Cell RNA sequencing and epigenetic landscaping of the BCE core locus in human and mouse craniofacial development.

a, UMAP clustering and *HMX1* expression profiling in early human embryonic craniofacial regions. scRNA-seq analysis showcases the UMAP clustering of cell types from the human early organogenesis atlas, with cell type annotations as per Xu, Y. *et al.* (2023)[1]. The central feature plot visualizes the expression levels of the *HMX1* gene across different cell clusters, while the right panel provides a detailed view of *HMX1* expression within craniofacial mesenchyme cells specifically in the pharyngeal arch (PA) structures. **b**, Conservation of epigenetic regulatory elements in mouse E8.5 NCC progenitors and E10.5 Md, PA2 NCCs. This panel illustrates the epigenetic landscape at the BCE core locus in mouse neural crest cell progenitors at E8.5 and E10.5 using publicly available datasets[2]. Highlighted in yellow are the regions orthologous to human enhancers and the *Hmx1* promoter. **c,** Dynamics of the BCE core locus epigenetic landscape from mouse pharyngeal arch 2 (PA2) to pinna development. Here, the evolution of the epigenetic context is traced from E10.5 PA2 to E14.5 pinna tissues using publicly available datasets[3]. Blue and red arcs indicate chromatin interactions from two Promoter Capture Hi-C (PC-HiC) dataset replicates. ATAC-seq and CTCF tracks are also shown.

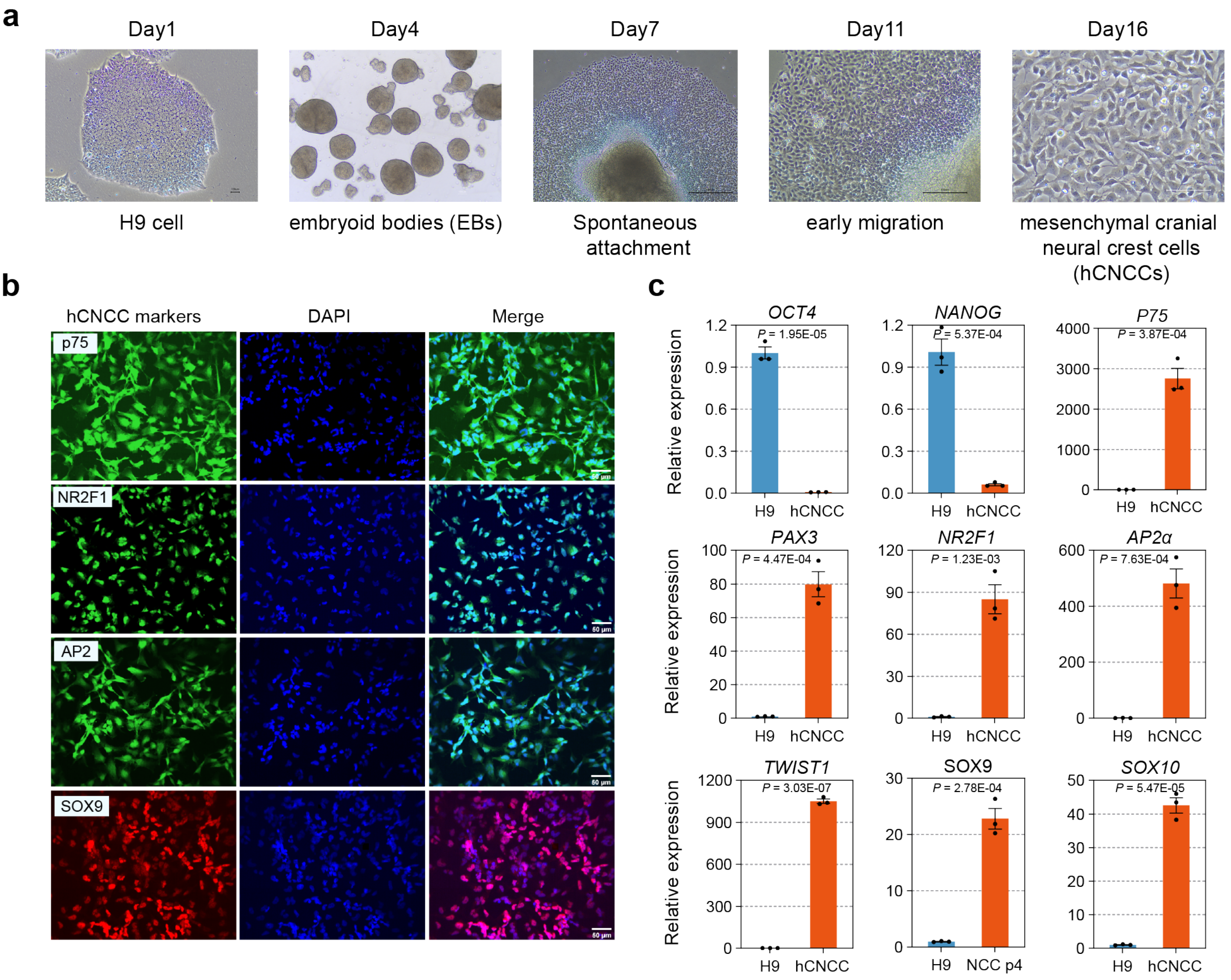

Supplementary Figure 3 | *In vitro* differentiation system from H9 cells to late hCNCCs state.

a, Representative images (three independent differentiations) depicting the progression from H9 cells to the late hCNCCs state. **b**, Immunofluorescence analysis using markers p75, NR2F1, AP2, and SOX9 to verify cell identity during the differentiation process. Three independent experiments were performed. **c**, Quantitative real-time PCR (qPCR) is used to further evaluate the efficiency of hCNCCs differentiation. Results are presented as mean ± SEM (n = 3). *P*-values were calculated using unpaired Student's *t*-test (two-tailed). Source data is provided as a Source Data file.

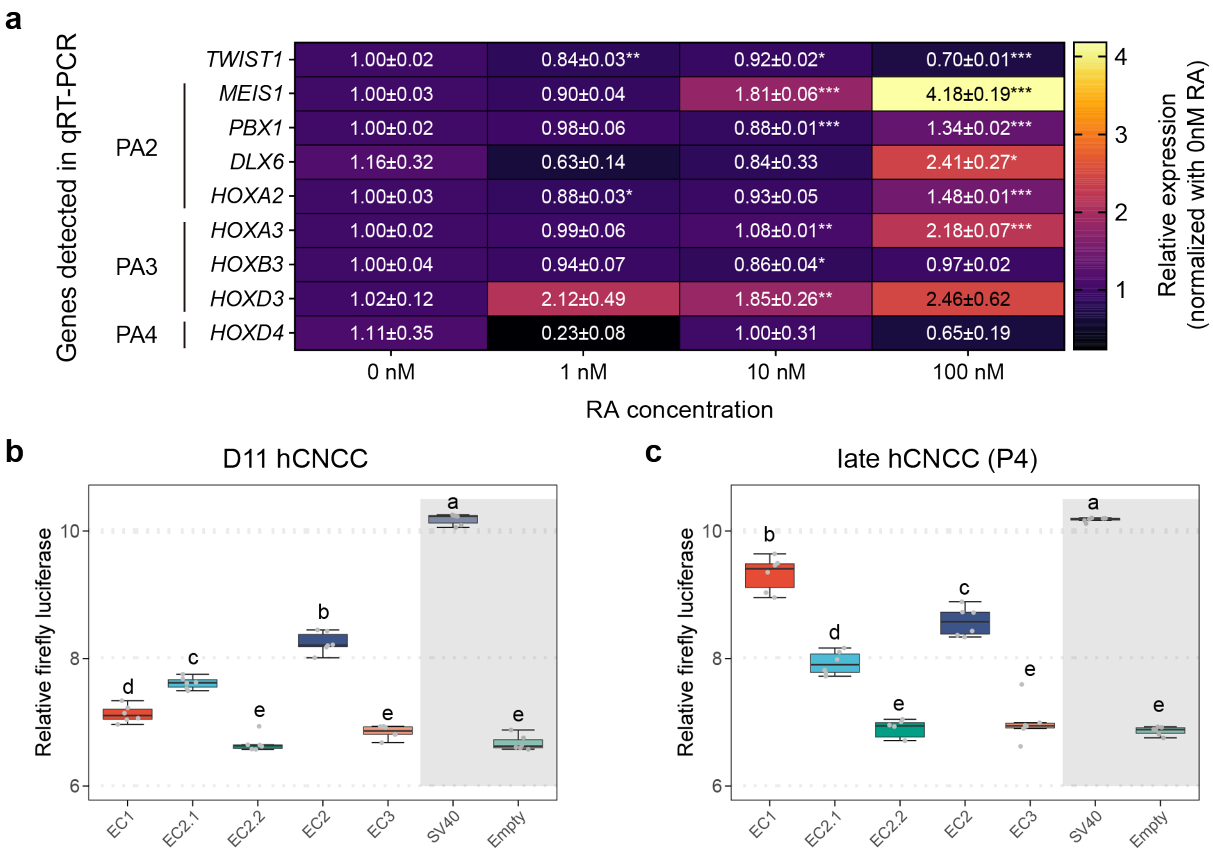

Supplementary Figure 4 | *In vitro* differentiation system from late hCNCCs to PA-like hCNCCs state.

a, qRT-PCR analysis of specifically expressed genes in PAs (PA2/3/4). Relative expressions of other RA-treated hCNCCs (1 nM, 10 nM, 100 nM) were normalized with 0 nM RA treatment hCNCCs. *P*-values were calculated using unpaired Student's *t*-test (two-tailed) compared with 0 nM RA treatment hCNCCs for each gene. Values are presented as mean ± S.E.M. (n = 4), **P*<0.05, ***P*<0.01, ****P*<0.001. **b**, **c**, Luciferase assays evaluating candidate enhancers at the BCE core locus in D11 hCNCCs (**b**), and late hCNCC (**c**), including SV40 enhancer (positive control) and empty vector (negative control). Results from two independent experiments, each with three technical replicates (n = 6), are shown. One-way ANOVA was performed using GraphPad, followed by Duncan's multiple comparison test. Groups sharing the same letter indicate no significant difference, while groups with different letters indicate significant differences. Source data is provided as a Source Data file.

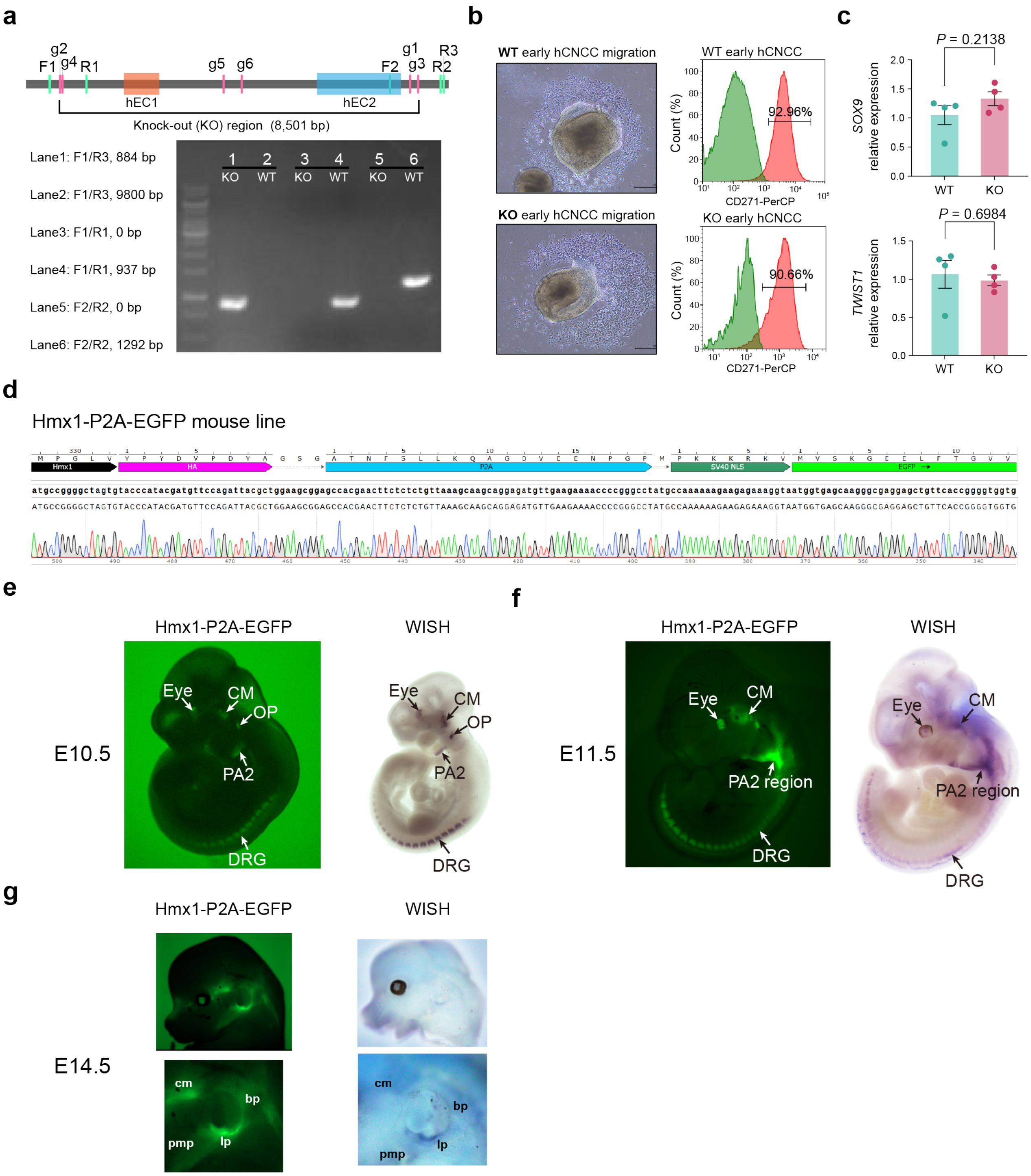

Supplementary Figure 5 | Validation of transgenic knock-out cell line and knock-in mouse line.

a, A CRISPR/Cas9 strategy is employed to delete the hEC1+hEC2 region (~9 kb). Six gRNA sequences are designed along with corresponding primer pairs to validate knockout efficiency, with gel analysis images provided. **b**, Representative images (three independent differentiations) illustrate early hCNCCs migration in both wild-type (WT) and knockout (KO) cell lines. Scale bars measure 100 µm. In the right panel, FACS analysis of CD271 (p75) in early hCNCCs is shown for both WT and KO cell lines. **c**, Gene expression analysis of *SOX9* and *TWIST1*, two important TFs in hCNCCs, compares WT and KO. Data are presented as mean ± SEM (n = 4). *P*-values were calculated using unpaired Student's *t*-test (two-tailed). Source data is provided as a Source Data file. **d**, Construction of the Hmx1-P2A-EGFP transgenic reporter line is described, with Sanger sequencing results of this mouse model presented. **e**, **f**, **g**, Comparison of the spatiotemporal pattern of *Hmx1* expression between the Hmx1-P2A-EGFP transgenic reporter line and *WISH* results at E10.5 (**e**), E11.5 (**f**) and E14.5 (**g**). The left panel shows an image of Hmx1-P2A-EGFP; the right panel shows an image of *WISH* result. CM, craniofacial mesenchymal; DRG, dorsal root ganglia; OP, otic placode; bp, basal pinna; lp, lower part of pinna; pmp, the proximal region of the mandibular prominence. Three independent experiments were performed.

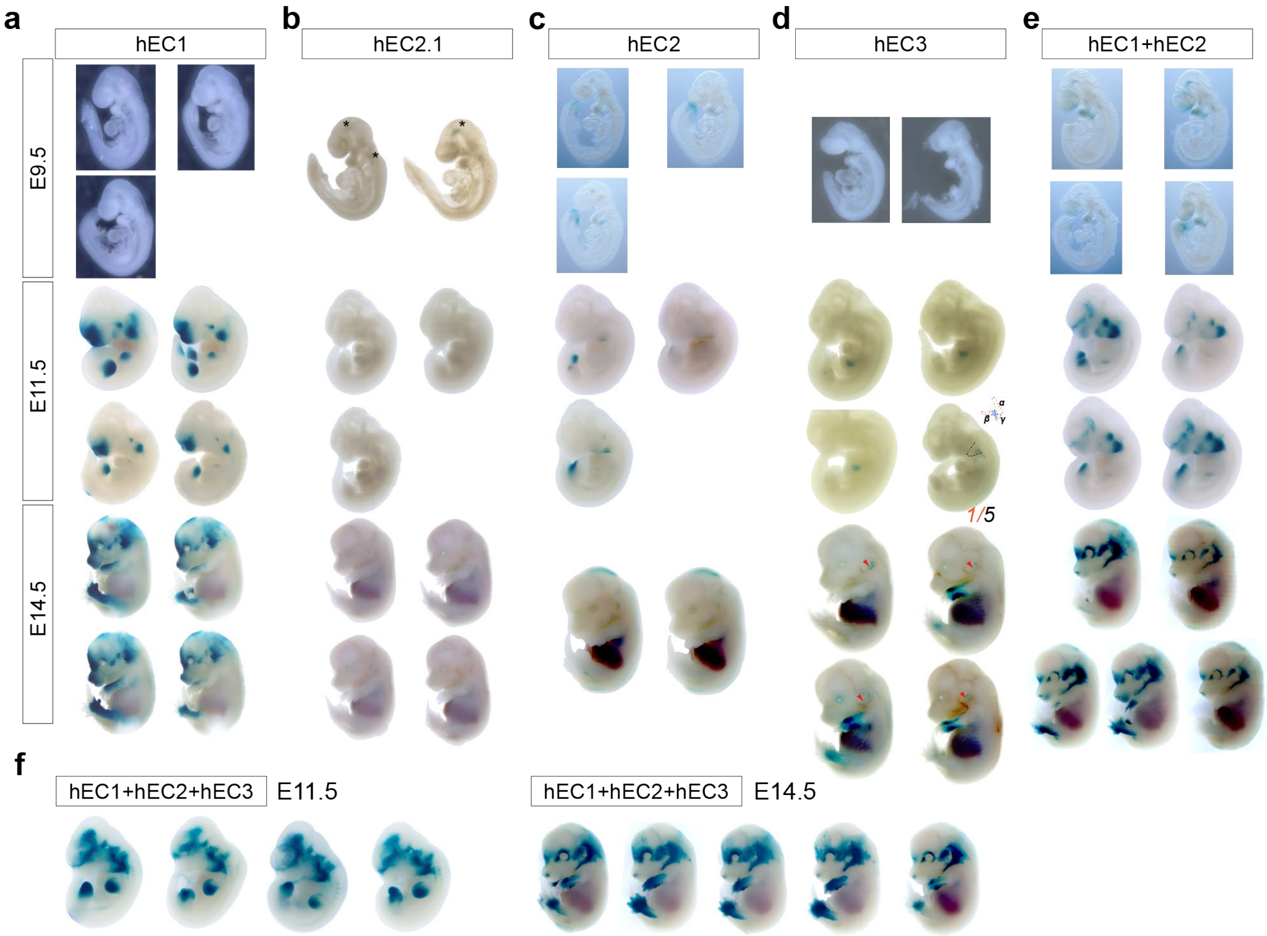

Supplementary Figure 6 | The activity of multipartite enhancers closely recapitulates the spatiotemporal expression pattern of endogenous *Hmx1*.

a-f, LacZ assays are conducted to track pinna development from E9.5 to E14.5 for various enhancers: hEC1 (**a**), hEC2.1 (**b**), hEC2 (**c**), hEC3 (**d**), hEC1+hEC2 (**e**) and hEC1+hEC2+hEC3 (**f**). Results showing all positive LacZ staining embryos for each enhancer are included.

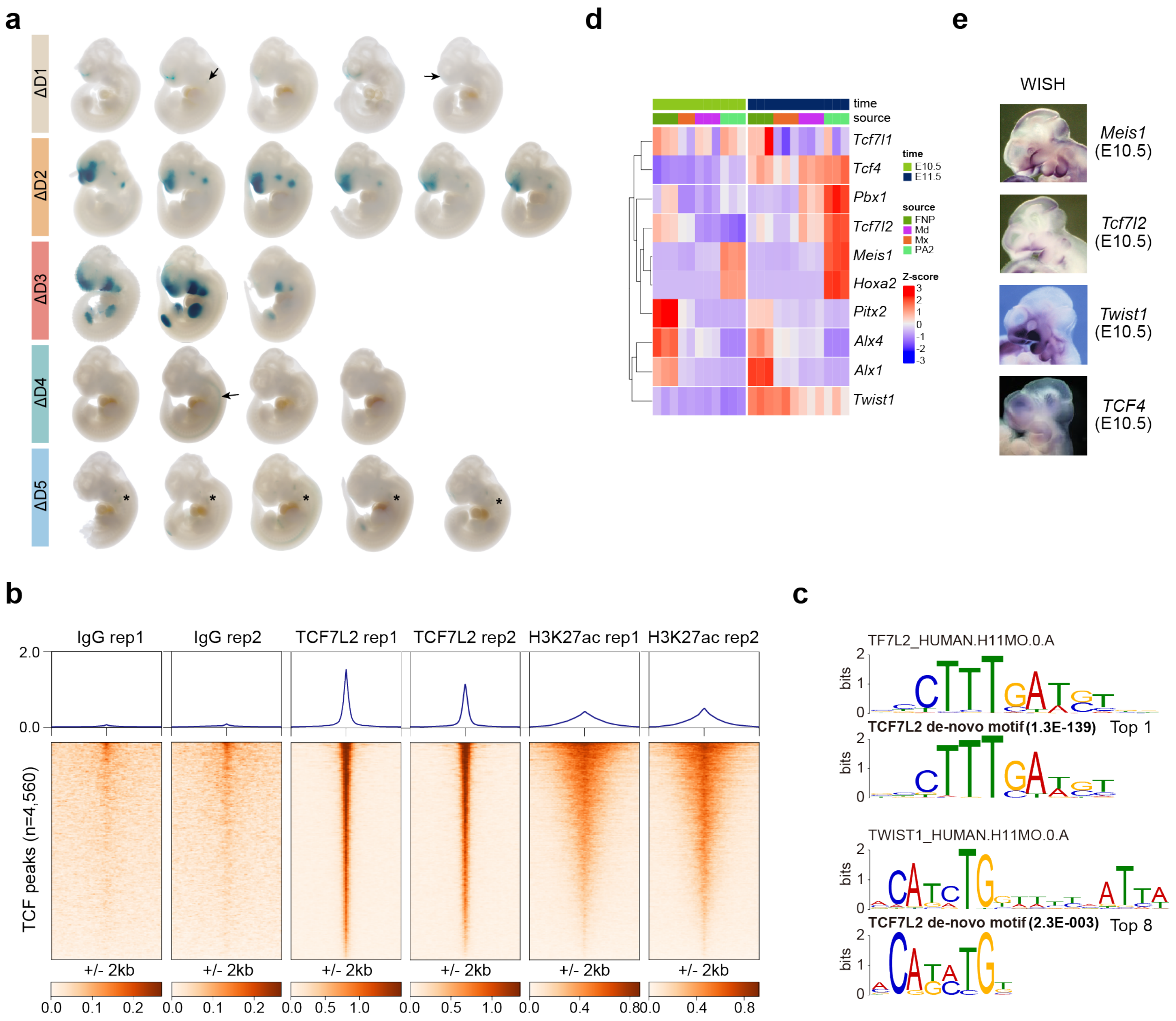

Supplementary Figure 7 | HD, HMG box and Coordinator TFs coordinately regulate hEC1 specificity and activity. **a,** Display all positive LacZ staining embryos for ΔD1, ΔD2, ΔD3, ΔD4, and ΔD5. Arrows point to faint staining signals. **b,** A heatmap is presented, showing IgG, H3K27ac, and TCF7L2 binding profiles across ±2 kb from TCF7L2 peak summits (4560). Results from two independent biological replicates are shown. **c,** *De novo* TCF7L2 motif analysis is performed using the MEME toolkit. The top-ranked motif is similar to TF7L2_HUMAN.H11MO.O.A; the eighth-ranked motif is similar to TWIST1_HUMAN.H11MO.O.A. **d,** A complex heatmap shows TFs expression across four craniofacial regional zones: frontonasal process (FNP), maxilla (Mx), mandible (Md), and PA2 at E10.5 and E11.5[2]. Gene expression is scaled and clustering is performed. **e,** *WISH* images of four critical TFs including *Meis1*, *Tcf7l2*, *Twist1* and *Tcf4*. Images are from Emage database (*https://www.emouseatlas.org/emage/home.php*).

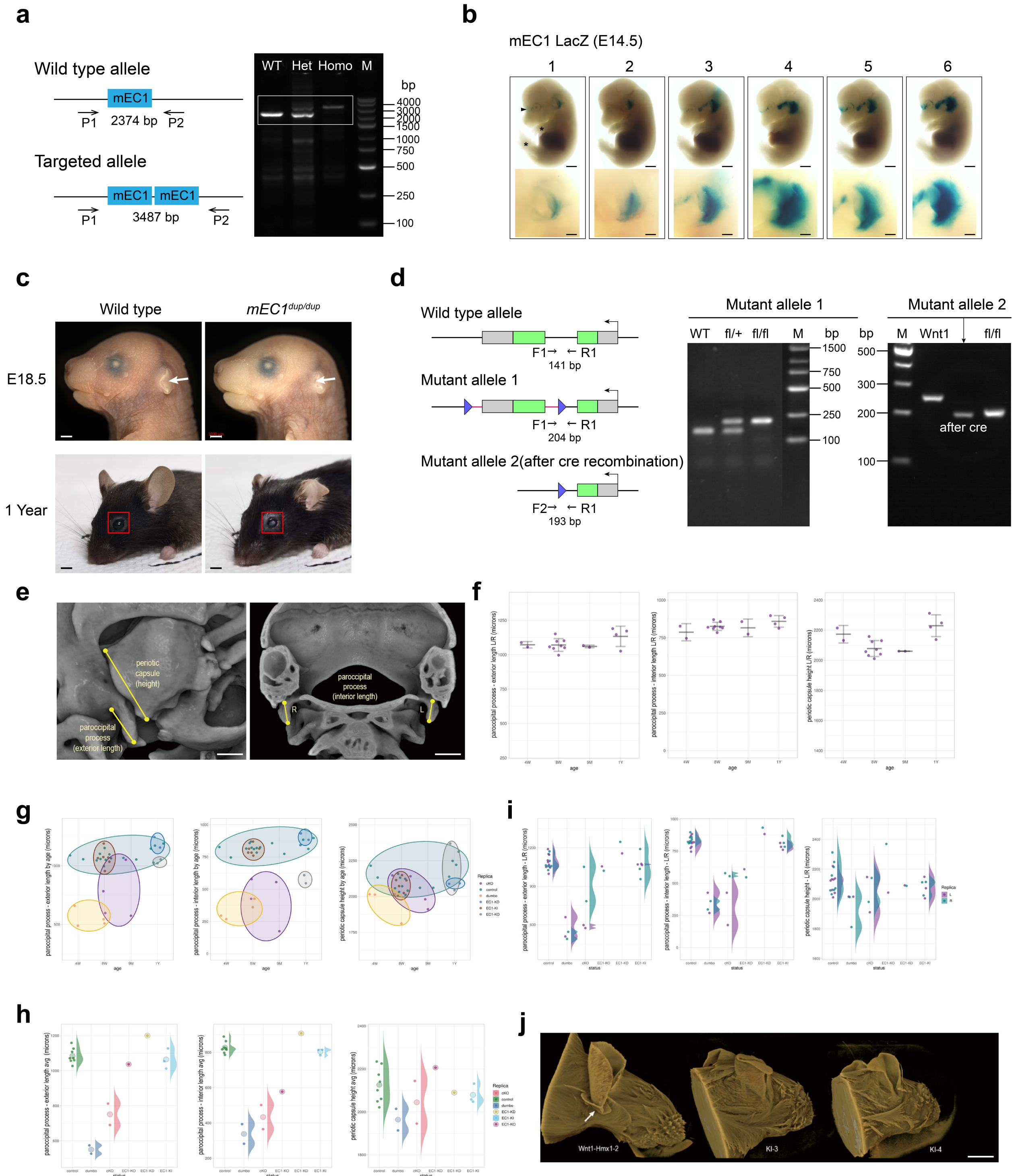

Supplementary Figure 8 | Pinna malformation is driven by either the expansion or the loss of spatial *Hmx1* expression. **a,** A schematic diagram of the transgenic *mEC1*$^{dup/dup}$ mouse model is presented. A primer pair, P1 and P2, flanking the mEC1 element, is designed for genotyping: the wildtype allele measures 2374 bp and the transgenic allele 3487 bp. The right panel displays gel electrophoresis results for wildtype (WT), heterozygote (Het), and homozygote (Homo) in the white rectangle. **b,** The figure Depicts all positive LacZ staining in mouse embryos for mEC1 at E14.5. The upper panel shows the whole embryo, while the bottom panel presents zoomed-in images of pinna staining. Black arrows and stars indicate distinct staining patterns compared to hEC1 LacZ results, particularly in the frontonasal prominence and fore- and hind-limbs. Scale bars measure 500 µm. **c,** Ear and eye malformations are compared between wildtype and *mEC1*$^{dup/dup}$ mice. Ear malformation in *mEC1*$^{dup/dup}$ is clearly visible, as indicated by the white arrow. Eye malformation, likely representing cataract, is marked with a red rectangle in older mice. Scale bars measure 1000 µm. **d,** A schematic diagram of the transgenic *wnt1::Cre;Hmx1*$^{fl/fl}$ mouse model is shown. Primers F1, R1 are used to identify WT and *Hmx1*$^{fl/fl}$; and F2, R1 for genotyping *Wnt1::Cre;Hmx1*$^{fl/fl}$. **e,** The exterior and interior length of each paroccipital process and the height of the periodic capsule on each side were measured on 3D rendered scans using the 3D coordinate landmarks denoted by the yellow dots. Scale bars measure 500 µm.

f, The exterior and interior lengths of the paroccipital processes and the height of the periotic capsule in control mice measured at 4 weeks, 8 weeks, 9 months and 1 year of age. The measurements suggest that the paroccipital processes have reached their near-final adult length by 4 weeks of age. **g,** Paroccipital process and periotic capsule measurements were graphed based on age, showing the distinct genotypes. **h**, The average of the left and right side paroccipital process measurements and the periotic capsule measurements were graphed to display the respective genotype-specific sizes. The dots represent separate measurements. The distribution of each genotype is also shown to the right of the individual measurements for each. **i**, Left and right side paroccipital process and periotic capsule measurements were graphed separately to assess lateral variability. Left side (purple dots/distribution), right side (teal dots/distribution) based on genotypes. The dots represent separate measurements. The distribution is shown to the right of the individual measurements for each side. **j,** 3D renderings of the soft tissue on heads of *Wnt1::Cre;Hmx1*$^{fl/fl}$ and *mEC1*$^{dup/dup}$ mice. White arrow indicates the pinna cartilage duplication, which is also seen in *dumbo* mice. Scale bars measure 500 µm. Source data is provided as a Source Data file.

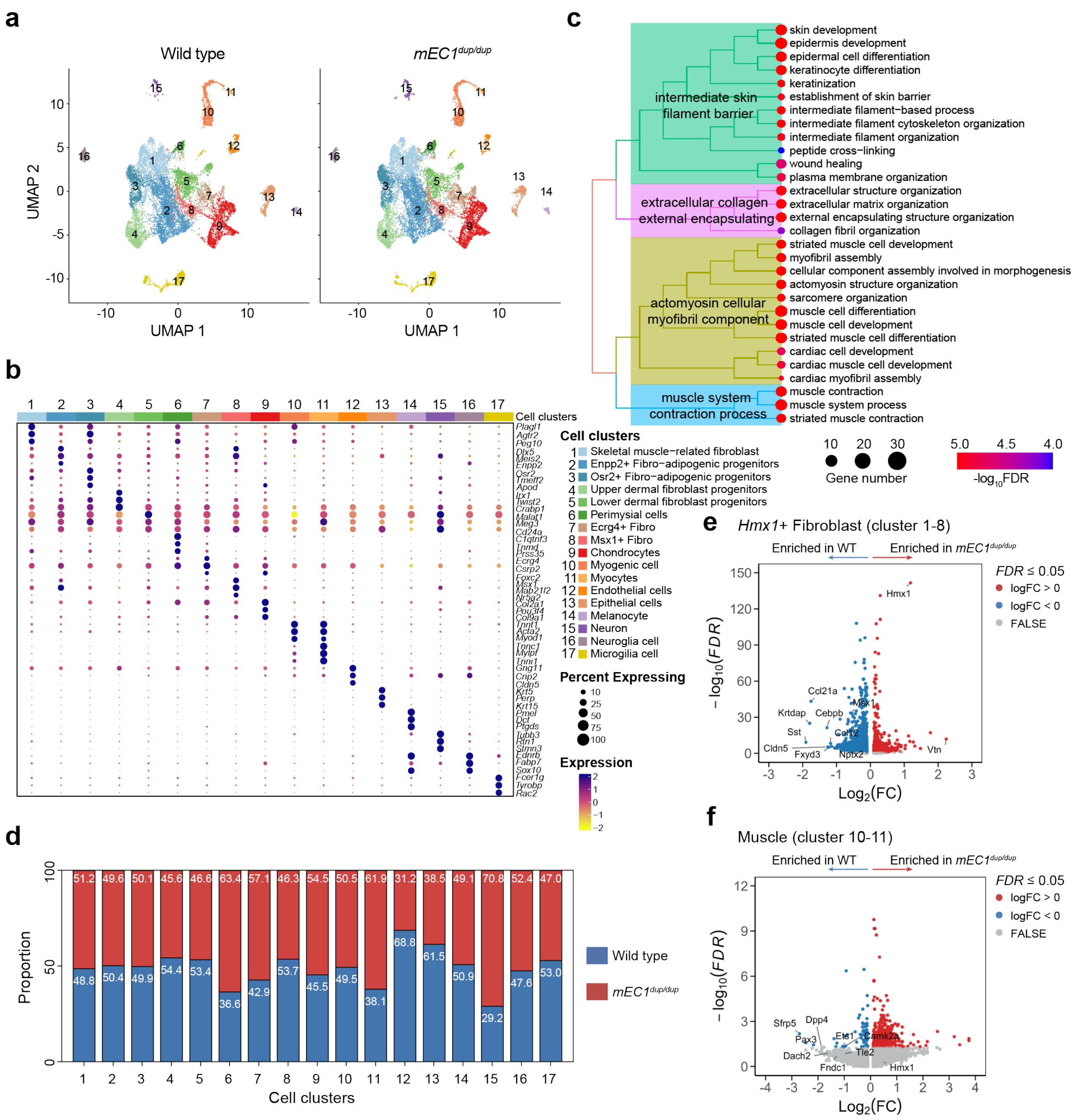

Supplementary Figure 9 | Bulk and ScRNA-seq reveals the disrupted gene network in fibroblasts, cartilage, muscle and epidermis.

a, UMAP dimensional reduction visualization of wild type and *mEC1*$^{dup/dup}$. **b**, Marker genes used for 17 cell cluster annotations are identified. The top 3 genes for each cell cluster are presented. **c**, Gene Ontology (GO) enrichment analysis of 284 downregulated genes detected in bulk RNA-seq is performed. The top 30 GO terms are displayed and grouped into four clusters. **d**, Cell type proportion of each cell cluster between wild type and *mEC1*$^{dup/dup}$. **e, f**, Single cell differentially gene analysis in fibroblasts (**e**) and muscle (**f**) cluster. DEGs are defined as FDR value ≤ 0.05 and |log2 FC| ≥ 0. Up- and down-regulated genes are shown as red and blue dots, respectively. Some genes that are critical for cell development are labeled. Source data is provided as a Source Data file.

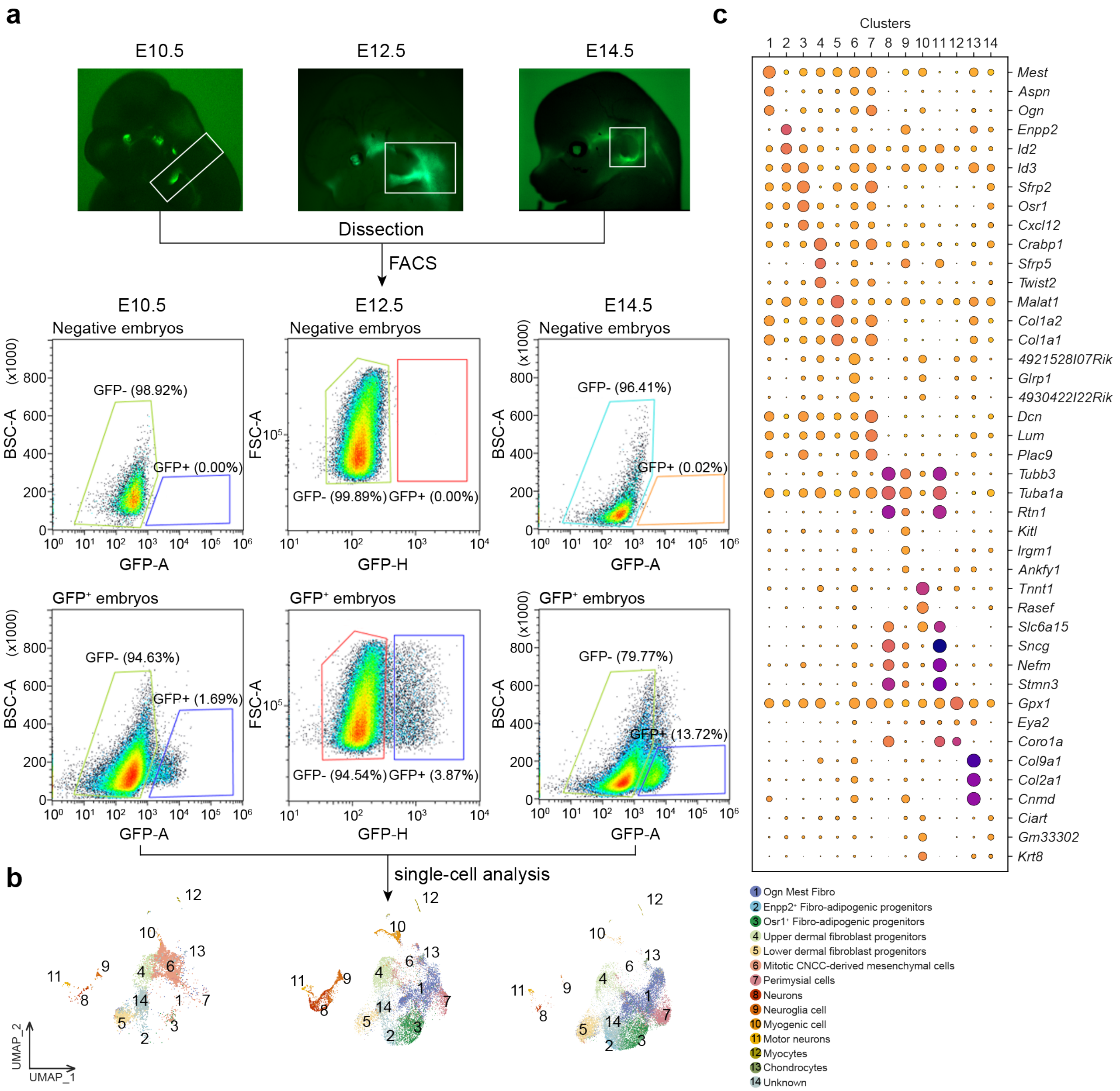

Supplementary Figure 10 | ScRNA-seq reveals the critical role of *Hmx1* on the ear fibroblast development.

a, Lineage tracing of *Hmx1*$^{+}$ cells from E10.5 to E14.5 using the Hmx1-P2A-EGFP mouse reporter line. Fluorescence images for each timepoint are shown, with tissues micro-dissected for dissociation marked by white rectangles. *GFP*$^{+}$ cells are collected using FACS. **b**, UMAP dimensional reduction visualization of *Hmx1*$^{+}$ expressing cells for each timepoint is presented. **c**, Marker genes used for cell cluster annotation are identified.

Supplementary references

1 Xu, Y. *et al.* A single-cell transcriptome atlas profiles early organogenesis in human embryos. *Nat Cell Biol* **25**, 604-615 (2023). https://doi.org/10.1038/s41556-023-01108-w

2 Minoux, M. *et al.* Gene bivalency at Polycomb domains regulates cranial neural crest positional identity. *Science* **355** (2017). https://doi.org/10.1126/science.aal2913

3 Kessler, S. *et al.* A multiple super-enhancer region establishes inter-TAD interactions and controls Hoxa function in cranial neural crest. *Nat Commun* **14**, 3242 (2023). https://doi.org/10.1038/s41467-023-38953-0

4 Vorontsov, I. E. *et al.* HOCOMOCO in 2024: a rebuild of the curated collection of binding models for human and mouse transcription factors. *Nucleic Acids Res* **52**, D154-D163 (2024). https://doi.org/10.1093/nar/gkad1077

5 Grant, C. E., Bailey, T. L. & Noble, W. S. FIMO: scanning for occurrences of a given motif. *Bioinformatics* **27**, 1017-1018 (2011). https://doi.org/10.1093/bioinformatics/btr064
