# Supplementary material for: Auricular malformations are driven by copy number variations in a hierarchical enhancer cluster and a dominant enhancer recapitulates human pathogenesis
Source: Nat Commun. 2025 May 17;16:4598. doi: 10.1038/s41467-025-59735-w (PMC12085581; doi:10.1038/s41467-025-59735-w)
Supplement: Supplementary file 2 — Description of Additional Supplementary Files [file 41467_2025_59735_MOESM2_ESM.pdf]

Supplementary Data 1: PC-HiC interaction information between *HMX1* promoter and other genomic regions.

Each row in the table contains the genomic locations (hg19) of two regions of the *HMX1* promoter and it's interacting locus, and the interaction score also shows.

Supplementary Data 2: Transcription factor binding sites (TFBSs) prediction using FIMO on the hEC1 sequence.

The table contains motif cluster, motif ID, position, TF symbol, TF class, TF family, score, and *p*-value and *q*-value for each motif (HOCOMOCO V12 database[4]) bound by TFs that are co-expressed with *HMX1* in craniofacial mesenchyme predicted by FIMO software[5] on the hEC1 sequence. The program uses a dynamic programming algorithm to convert log-odds scores into *p*-values, assuming a zero-order background model, and *q*-values were calculated by Benjamini-Hochberg (BH) method. Five clusters of motifs were divided based on the distribution density of motifs, termed as D1 (1~162 bp), D2 (152~292 bp), D3 (282~463 bp), D4 (453~630 bp) and D5 (620~801 bp). The novel coordinator pattern consisting of HD, HMG-box, Coordinator motif in D1 and D3-4 region are highlighted.

Supplementary Data 3: Results of identified protein binding to hEC1 using LC-MS.

The table contains three sheets: Negative control data, sample data, sample-NC data. Protein information identified in the DNA-pull down assay and subsequent LC-MS analysis were showed. And the table is sorted by the score for each protein, and Meis1, Pbx1/3, Dlx6 and Hmga2 TFs were highlighted.

Supplementary Data 4: EChO analysis results of TCF7L2 CUT&RUN on hCNCCs.

The table contains information on each position and its corresponding fragment size on the hEC1.

Supplementary Data 5: Differential gene expression analysis.

The table provides the information of differential expression genes (DEGs) between $mEC1^{dup/dup}$ and Wild-type (WT) pinna structure at E14.5 mouse embryo. Five columns: Ensembl gene ID, gene symbol, log2 fold change (logFC), mean expression level (logCPM), false discovery rate (FDR),

and down- or up-regulated categories (group) in *mEC1*$^{dup/dup}$ compared with WT. See Methods for details on statistical analysis.

Supplementary Data 6: Motif enrichment analysis

The table contains motif enrichment analysis results of promoters and cis-regulatory elements (cREs) related to DEGs detected in bulk RNA-seq. Motif databases is HOCOMOCO V12 core database and simple enrichment analysis (SEA) in the meme software suite was performed. *P*-values are calculated by Fisher exact test, *E*-values are the *P*-values multiplied by the number of motifs and *Q*-values are calculated by Benjamini-Hochberg (BH) method.

Supplementary Data 7: GO enrichment analysis.

The table contains GO enrichment results of each GO ID, description, GeneRatio, Background ratio (BgRatio), p value (calculated by hypergeometric distribution), p.adjust (adjusted by Benjamini-Hochberg, BH), q value (adjusted by Storey Q value method), geneID and gene count (Count).

Supplementary Data 8: Primer lists used in this paper.

The table contains four sheets: RT-PCR primer, LUC primer, LacZ primer and Genotyping primer. RT-PCR primer sheet contains primers for gene expression analysis; LUC primer sheet contains primers of constructing luciferase-related vectors for cell transfection; LacZ primer sheet contains primers for constructing enSERT assay-related vectors; Genotyping primer sheet contains primers for transgenic cell line and mouse models genotyping and validation.

Supplementary Data 9: Probe sequence information used in EMSA and in-situ RNA hybridization.

DNA sequence information used in EMSA and WISH are provided.
