# Supplementary material for: Auricular malformations are driven by copy number variations in a hierarchical enhancer cluster and a dominant enhancer recapitulates human pathogenesis
Source: Nat Commun. 2025 May 17;16:4598. doi: 10.1038/s41467-025-59735-w (PMC12085581; doi:10.1038/s41467-025-59735-w)
Supplement: Supplementary file 12 — Reporting Summary [file 41467_2025_59735_MOESM12_ESM.pdf]

nature portfolio

Corresponding author(s): Yong-Biao Zhang

Last updated by author(s): Mar 28, 2025

Reporting Summary

Nature Portfolio wishes to improve the reproducibility of the work that we publish. This form provides structure for consistency and transparency in reporting. For further information on Nature Portfolio policies, see our Editorial Policies and the Editorial Policy Checklist.

Statistics

For all statistical analyses, confirm that the following items are present in the figure legend, table legend, main text, or Methods section.

n/a	Confirmed	
☐	☒	The exact sample size (*n*) for each experimental group/condition, given as a discrete number and unit of measurement
☐	☒	A statement on whether measurements were taken from distinct samples or whether the same sample was measured repeatedly
☐	☒	The statistical test(s) used AND whether they are one- or two-sided<br>*Only common tests should be described solely by name; describe more complex techniques in the Methods section.*
☒	☐	A description of all covariates tested
☐	☒	A description of any assumptions or corrections, such as tests of normality and adjustment for multiple comparisons
☐	☒	A full description of the statistical parameters including central tendency (e.g. means) or other basic estimates (e.g. regression coefficient) AND variation (e.g. standard deviation) or associated estimates of uncertainty (e.g. confidence intervals)
☐	☒	For null hypothesis testing, the test statistic (e.g. *F*, *t*, *r*) with confidence intervals, effect sizes, degrees of freedom and *P* value noted<br>*Give P values as exact values whenever suitable.*
☒	☐	For Bayesian analysis, information on the choice of priors and Markov chain Monte Carlo settings
☒	☐	For hierarchical and complex designs, identification of the appropriate level for tests and full reporting of outcomes
☒	☐	Estimates of effect sizes (e.g. Cohen's *d*, Pearson's *r*), indicating how they were calculated

Our web collection on statistics for biologists contains articles on many of the points above.

Software and code

Policy information about availability of computer code

Data collection	Illumina HiSeq X10 system, Illumina NovaSeq 6000,
Data analysis	CNV analysis:<br>GenomeStudio (v2011.1), FastQC (v1.1.0), Cutadapt (v1.15), BWA (v0.7.16a), Picard (v2.27.0), Samtools (v1.18), GATK (v4), MERLIN (v.1.1.2), Plink (v1.9), cn.mops (v1.48.0)<br><br>RNA-seq analysis:<br>FastQC (v0.12.1), STAR (v2.7.11a), featureCounts (v2.0.6), limma (v3.56.2), Glimma (v2.10.0), edgeR (v3.42.4), clusterProfiler (v4.8.3), Mus.musculus (v1.3.1), org.Mm.eg.db (v3.17.0), dplyr (v1.1.3), ggplot2 (v3.4.3), ggrepel (v0.9.3), ggnewscale (0.4.9), DOSE (v3.26.2), enrichplot (v1.20.3), tidyr (v1.3.0), RColorBrewer (v1.1-3)<br><br>ATAC-seq, ChIP-seq, CUT&RUN analysis:<br>Samtools (version 1.3.1), MACS2 (version 2.2.7.1), bedtools (version 2.31.0), deeptools (version 3.5.2), R (version 3.6.3), Python (version 3.6.15), Perl (version v5.32.1), Java (openjdk version "1.8.0_292", OpenJDK Runtime Environment (build 1.8.0_292-b10), OpenJDK 64-Bit Server VM (build 25.292-b10, mixed mode)), picard (version 2.8.0), meme (version 5.1.1), tabix (version 1.11), Genome (GRCh38.p14), pyGenomeTracks (version 3.8)<br><br>HiC and PC-HiC analysis:<br>hicup_digester, HiCUP software (version 0.9.2), Chicago R package (version 1.30.0), bam2chicago.sh, makeDesignFiles.py, pygenometrack (version 3.9), HiCExplorer (version 3.7.2)

scRNA-seq analysis:
CeleScope pipeline (https://github.com/singleron-RD/CeleScope), Seurat (version 4.3.0), SCANPY (version 1.9.3), CellRank package (version 2.0.2)

EChO analysis:
EChO software (https://github.com/FredHutch/EChO)

Other software:
GraphPad Prism (v10)

For manuscripts utilizing custom algorithms or software that are central to the research but not yet described in published literature, software must be made available to editors and reviewers. We strongly encourage code deposition in a community repository (e.g. GitHub). See the Nature Portfolio guidelines for submitting code & software for further information.

Data

Policy information about availability of data

All manuscripts must include a data availability statement. This statement should provide the following information, where applicable:

- Accession codes, unique identifiers, or web links for publicly available datasets
- A description of any restrictions on data availability
- For clinical datasets or third party data, please ensure that the statement adheres to our policy

All raw sequencing data and processed data in this study have been deposited in the NCBI Gene Expression Omnibus (GEO) database under accession code GSE263084 (CUT&RUN, https://www.ncbi.nlm.nih.gov/geo/query/acc.cgi?acc=GSE263084), GSE263085 (PC-HiC, https://www.ncbi.nlm.nih.gov/geo/query/acc.cgi?acc=GSE263085), GSE263086 (bulk RNA-seq, https://www.ncbi.nlm.nih.gov/geo/query/acc.cgi?acc=GSE263086), GSE263087 (scRNA-seq, https://www.ncbi.nlm.nih.gov/geo/query/acc.cgi?acc=GSE263087). The raw genomic data are available under restricted access for patients' confidentiality, access requires a brief project description and a signed data-use agreement restricting downstream data sharing and limiting use to the requesting investigator. In addition, we used public sequencing datasets: ChIP-seq datasets: GSE28874 (d11hCNCC, https://www.ncbi.nlm.nih.gov/geo/query/acc.cgi?acc=GSE28874), GSE145327 (P4hCNCC, https://www.ncbi.nlm.nih.gov/geo/query/acc.cgi?acc=GSE145327), GSE70751 (P4hCNCC, https://www.ncbi.nlm.nih.gov/geo/query/acc.cgi?acc= GSE70751), GSE89435 (mCNCC, https://www.ncbi.nlm.nih.gov/geo/query/acc.cgi?acc= GSE89435), GSE211900 (Pinna, https://www.ncbi.nlm.nih.gov/geo/query/acc.cgi?acc= GSE211900). ATAC-seq datasets: GSE108517 (d11hCNCC, https://www.ncbi.nlm.nih.gov/geo/query/acc.cgi?acc= GSE108517), GSE145327 (hESC and P4hCNCC, https://www.ncbi.nlm.nih.gov/geo/query/acc.cgi?acc= GSE145327), GSE89436 (mCNCC, https://www.ncbi.nlm.nih.gov/geo/query/acc.cgi?acc= GSE89436), GSE211899 (Pinna, https://www.ncbi.nlm.nih.gov/geo/query/acc.cgi?acc= GSE211899). scRNA-seq dataset: GSE157329 (single-cell transcriptomes of 4- to 6-week human embryos, https://www.ncbi.nlm.nih.gov/geo/query/acc.cgi?acc= GSE157329). Additionally, human embryonic craniofacial epigenetic data from stages CS13, CS14, CS15, and CS17 were accessed from https://cotney.research.uchc.edu/craniofacial/. Source data are provided with this paper.

Research involving human participants, their data, or biological material

Policy information about studies with human participants or human data. See also policy information about sex, gender (identity/presentation), and sexual orientation and race, ethnicity and racism.

Reporting on sex and gender	Sex and gender data have been collected. No sex- and gender-based analyses have been performed, as patients with bilateral constricted ear (BCE) show no sex differences, and gender differences in our mouse model do not impact disease occurrence.
Reporting on race, ethnicity, or other socially relevant groupings	The population involved in this study was Han Chinese from China.
Population characteristics	The populations characteristics with bilateral constricted ear are of Chinese ancestry as described in the manuscript
Recruitment	The cohort of patients with bilateral constricted ear (BCE) were recruited in genetics clinics on the basis of clinical findings compatible with the clinical diagnosis of BCE.
Ethics oversight	For this study, written informed consent for participation in genetic and biological research was obtained from all subjects or their legal guardians. Ethical approval was granted by the Ethics Committees of the School of Biological Science and Medicine Engineering at Beihang University and the Plastic Surgery Hospital of the Chinese Academy of Medical Sciences. The study has been registered and approved by China's Ministry of Science and Technology (project 2023-CJ0849).

Note that full information on the approval of the study protocol must also be provided in the manuscript.

Field-specific reporting

Please select the one below that is the best fit for your research. If you are not sure, read the appropriate sections before making your selection.

☒ Life sciences ☐ Behavioural & social sciences ☐ Ecological, evolutionary & environmental sciences

For a reference copy of the document with all sections, see nature.com/documents/nr-reporting-summary-flat.pdf

Life sciences study design

All studies must disclose on these points even when the disclosure is negative.

Sample size	For clinical sample size, we collected a cohort of seven Chinese families (from five separate provinces) and 30 BCE patients, 16 normal people in the corresponding families were sequenced. This sample size is the largest reported in the biomedical literature.<br>For sequencing size, we chose samples sizes regarding replicates based on the standards of the field (the ENCODE Data standards).<br>For experimental size, we chose samples sizes regarding replicates based on the sensitivity of corresponding experimental types.<br>We did not perform any statistical tests to predetermine sample sizes.
Data exclusions	No data were excluded
Replication	For qPCR and histological experiments, all the experiments are based on at least three biologically independent replicates.<br>For luciferase assay, experiments are based on at least two or three biologically independent cell differentiation processes, and 2~4 technical replicates were performed at each biologically replicates.<br>For in vivo LacZ assay, staining results are reported based on at least three positive embryos except for the hEC3 staining pattern (one patten only have one replicate may be caused by the very low intrinsic enhancer activity in pinna zone).<br>For RNA-seq experiments, five independent embryos for wild type and mEC1dup/dup mice embryos were included.<br>For CUT&RUN assay, two biological replicates were included.<br>Overall, reproducibility among replicates for all experiments was high.<br>For scRNA-seq of lineage tracing in mouse embryo pinna and promoter-capture HiC in-vitro hCNCCs, one replicate was generated in this study.
Randomization	In the mouse studies, biological replicates were derived from siblings from separate litters. The mice and embryos utilized were bred and/or set up identically, and were distributed randomly across various experimental categories.
Blinding	There was not blinding in this rather diagnostic study

Reporting for specific materials, systems and methods

We require information from authors about some types of materials, experimental systems and methods used in many studies. Here, indicate whether each material, system or method listed is relevant to your study. If you are not sure if a list item applies to your research, read the appropriate section before selecting a response.

Materials & experimental systems

n/a	Involved in the study	
☐	☒	Antibodies
☐	☒	Eukaryotic cell lines
☒	☐	Palaeontology and archaeology
☐	☒	Animals and other organisms
☒	☐	Clinical data
☒	☐	Dual use research of concern
☒	☐	Plants

Methods

n/a	Involved in the study	
☐	☒	ChIP-seq
☐	☒	Flow cytometry
☒	☐	MRI-based neuroimaging

Antibodies

Antibodies used	Primary antibodies:<br>Mouse monoclonal anti-p75 antibody, Abcam, ab245134<br>Rabbit monoclonal anti-NR2F1 antibody, Abcam, ab181137<br>Mouse monoclonal anti-TFAP2A antibody, Santa Cruz, sc-12726<br>Rabbit polyclonal anti-SOX9 antibody, Millipore, AB5535<br>Rabbit polyclonal IgG antibody, EpiCypher, 13-0042k<br>Rabbit polyclonal anti-H3K27ac antibody, Active Motif, 39133<br>Rabbit polyclonal anti-TCF7L2 antibody, Cell Signaling, 2569S<br><br>Secondary antibodies:<br>Goat polyclonal anti-mouse Alexa Fluor 488 antibody, Thermo fisher, A28175<br>Goat polyclonal anti-rabbit Alexa Fluor 594 antibody, Thermo fisher, A-11012<br>Mouse monoclonal PerCP/Cyanine5.5 anti-human CD271 (NGFR) antibody (ME20.4), BioLegend, 345112
Validation	Primary antibodies:<br>Mouse monoclonal anti-p75 antibody, Abcam, ab245134: validated by the vendor by WB, Flow Cyt, ICC/IF, IP<br>Rabbit monoclonal anti-NR2F1 antibody, Abcam, ab181137: validated by the vendor by Flow Cyt (Intra), WB, IHC-P, ICC/IF<br>Mouse monoclonal anti-TFAP2A antibody, Santa Cruz, sc-12726: validated by the vendor by WB, IP, IF and IHC(P)<br>Rabbit polyclonal anti-SOX9 antibody, Millipore, AB5535, Abcam, ab181137: validated by the vendor by IHC, WB, ChIP, ChIP-seq, ICC, IF<br>Rabbit polyclonal IgG antibody, EpiCypher, 13-0042k: validated by the vendor by CUT&RUN, CUT&Tag

Rabbit polyclonal anti-H3K27ac antibody, Active Motif, 39133: validated by the vendor by ChIP, ChIP-Seq, ICC/IF, WB and CUT&Tag
Rabbit polyclonal anti-TCF7L2 antibody, Cell Signaling, 2569S: validated by the vendor by WB, Immunoprecipitation, CUT&RUN, CUT&Tag

Secondary antibodies:
Goat polyclonal anti-mouse Alexa Fluor 488 antibody, Thermo fisher, A28175: validated by the vendor by WB, IHC (F), ICC/IF, Flow Cytometry
Goat polyclonal anti-rabbit Alexa Fluor 594 antibody, Thermo fisher, A-11012: validated by the vendor by ICC/IF, Flow Cytometry
Mouse monoclonal PerCP/Cyanine5.5 anti-human CD271 (NGFR) antibody (ME20.4), BioLegend, 345112: validated by the vendor by Flow Cytometry

Eukaryotic cell lines

Policy information about cell lines and Sex and Gender in Research

Cell line source(s)	The human embryonic stem cell line (H9) was provided by UBIGENE (Guangzhou, China)
Authentication	The H9 cell line was authenticated by UBIGENE (Guangzhou, China)
Mycoplasma contamination	Cell lines were tested negative for Mycoplasma
Commonly misidentified lines (See ICLAC register)	No commonly misidentified lines were used.

Animals and other research organisms

Policy information about studies involving animals; ARRIVE guidelines recommended for reporting animal research, and Sex and Gender in Research

Laboratory animals

Mouse C57BL6 strain was used for generating different transgenic pinna malformation models, embryos in E9.5, E10.5 and E14.5 were collected for downstream experiment (in-situ hybridization, RNA-seq, etc.). For breeding, all mice used were at least 8 weeks old and not older than 8 month.

Mouse FVB strain was used for generating H11-integrated transgenic enhancer LacZ assay, embryos in E9.5, E11.5 and E14.5 were collected for LacZ staining.

All mice were housed in a temperature-controlled environment (20 ± 2°C) with regulated humidity (50-60%) and standard 12-hour light/dark photoperiod. Animals had continuous access to food and water throughout the experimental period.

Wild animals	The study did not involve wild animals
Reporting on sex	There are no sex differences in the resulting phenotypes
Field-collected samples	No involvement of field-collected samples
Ethics oversight	All animal experiments were approved by the Animal Care Committee in Beihang University

Note that full information on the approval of the study protocol must also be provided in the manuscript.

Plants

Seed stocks	*Report on the source of all seed stocks or other plant material used. If applicable, state the seed stock centre and catalogue number. If plant specimens were collected from the field, describe the collection location, date and sampling procedures.*
Novel plant genotypes	*Describe the methods by which all novel plant genotypes were produced. This includes those generated by transgenic approaches, gene editing, chemical/radiation-based mutagenesis and hybridization. For transgenic lines, describe the transformation method, the number of independent lines analyzed and the generation upon which experiments were performed. For gene-edited lines, describe the editor used, the endogenous sequence targeted for editing, the targeting guide RNA sequence (if applicable) and how the editor was applied.*
Authentication	*Describe any authentication procedures for each seed stock used or novel genotype generated. Describe any experiments used to assess the effect of a mutation and, where applicable, how potential secondary effects (e.g. second site T-DNA insertions, mosiacism, off-target gene editing) were examined.*

ChIP-seq

Data deposition

☒ Confirm that both raw and final processed data have been deposited in a public database such as GEO.

☒ Confirm that you have deposited or provided access to graph files (e.g. BED files) for the called peaks.

Data access links<br>*May remain private before publication.*	Related data have been uploaded to GEO database. GSE263084 (CUT&RUN, https://www.ncbi.nlm.nih.gov/geo/query/acc.cgi?acc=GSE263084)
Files in database submission	fastq and bigwig files for the following samples:<br>IgG_hCNCCs_rep1<br>IgG_hCNCCs_rep2<br>H3K27ac_hCNCCs_rep1<br>H3K27ac_hCNCCs_rep2<br>TCF7L2_hCNCCs_rep1<br>TCF7L2_hCNCCs_rep2
Genome browser session<br>(e.g. UCSC)	bigwig files used for figures are deposited in GEO. Genome browser views were generated with pyGenomeTracks (version 3.8)

Methodology

Replicates	Two biological replicates were performed in all CUT&RUN results
Sequencing depth	CUT&RUN sequencing libraries were sequenced on the Illumina NovaSeq 6000 platform (2 × 150 bp, paired-end). The total sequencing depth for each sample was between 8M to 12M reads.
Antibodies	Rabbit polyclonal IgG antibody, EpiCypher, 13-0042k<br>Rabbit polyclonal anti-H3K27ac antibody, Active Motif, 39133<br>Rabbit polyclonal anti-TCF7L2 antibody, Cell Signaling, 2569S
Peak calling parameters	CUT&RUN sequencing data were analyzed employing the CUT&RUNTools 2.0 pipeline (https://github.com/fl-yu/CUT-RUNTools-2.0). For histone peak calling, the fragment size filter was deactivated, while for TFs, fragments larger than 120 bp were filtered out. Normalized bigwig files were generated using an alignment scale factor based on spike-in reads from E. coli K12 strain MG1655
Data quality	All samples were quality controlled by the log report function in each procedure in the pipeline.
Software	All the software used are integrated into the CUT&RUNTools 2.0 pipeline, with the corresponding software as follows: bowtie2 (version 2.5.1), Samtools (version 1.3.1), MACS2 (version 2.2.7.1), bedtools (version 2.31.0), deeptools (version 3.5.2), R (version 3.6.3), Python (version 3.6.15), Perl (version v5.32.1), Java (openjdk version "1.8.0_292", OpenJDK Runtime Environment (build 1.8.0_292-b10), OpenJDK 64-Bit Server VM (build 25.292-b10, mixed mode)), picard (version 2.8.0), meme (version 5.1.1), tabix (version 1.11), Genome (GRCh38.p14), pyGenomeTracks (version 3.8)

Flow Cytometry

Plots

Confirm that:

☒ The axis labels state the marker and fluorochrome used (e.g. CD4-FITC).

☒ The axis scales are clearly visible. Include numbers along axes only for bottom left plot of group (a 'group' is an analysis of identical markers).

☒ All plots are contour plots with outliers or pseudocolor plots.

☒ A numerical value for number of cells or percentage (with statistics) is provided.

Methodology

Sample preparation	Micro-dissected forming pinna prominences from Hmx1wt/EGFP embryos were enzymatically dissociated using 0.5% trypsin/1× EDTA for 10 minutes at 37 °C (for E10.5 and E12.5 stages) and papain digestion mix for 7 minutes (for E14.5 stage). The treated tissues were then rinsed in ice-cold 1× DMEM, filtered, and GFP+ cells were enriched using FACS
Instrument	Sony MA900
Software	MA Cell Sorter Software
Cell population abundance	GFP positive cells were determined by comparing against equivalent tissues from negative control embryos which don't express GFP markers. Negative control embryos were from the same litter as positive embryos.
Gating strategy	Firstly, cells were selected by scatter areas (FSC-A vs BSC-A). Then doublets were excluded by assessing scatter areas (FSC-A vs FSC-H). At last, positive GFP cells were sorted based on fluorescent signals on 488 nm.

☒ Tick this box to confirm that a figure exemplifying the gating strategy is provided in the Supplementary Information.
